# Efficacy Evaluation of Thymosin Alpha 1 in Non-severe Patients With COVID-19: A Retrospective Cohort Study Based on Propensity Score Matching

**DOI:** 10.3389/fmed.2021.664776

**Published:** 2021-04-23

**Authors:** ChenLu Huang, Ling Fei, Wei Xu, WeiXia Li, XuDong Xie, Qiang Li, Liang Chen

**Affiliations:** Department of Liver Diseases, Shanghai Public Health Clinical Center, Fudan University, Shanghai, China

**Keywords:** severe acute respiratory syndrome coronavirus 2, coronavirus disease 2019, Thymosin alpha 1, Thymosin-α1, efficacy evaluation

## Abstract

**Objective:** Thymosin alpha 1 (Thymosin-α1) is a potential treatment for patients with COVID-19. We aimed to determine the effect of Thymosin-α1 in non-severe patients with COVID-19.

**Methods:** We retrospectively enrolled 1,388 non-severe patients with COVID-19. The primary and secondary clinical outcomes were evaluated with comparisons between patients treated with or without Thymosin-α1 therapy.

**Results:** Among 1,388 enrolled patients, 232 patients (16.7%) received both Thymosin-α1 therapy and standard therapy (Thymosin-α1 group), and 1,156 patients (83.3%) received standard therapy (control group). After propensity score matching (1:1 ratio), baseline characteristics were well-balanced between the Thymosin-α1 group and control group. The proportion of patients that progressed to severe COVID-19 is 2.17% for the Thymosin-α1 group and 2.71% for the control group (*p* = 0.736). The COVID-19-related mortality is 0.54% for the Thymosin-α1 group and 0 for the control group (*p* = 0.317). Compared with the control group, the Thymosin-α1 group had significantly shorter SARS-CoV-2 RNA shedding duration (13 vs. 16 days, *p* = 0.025) and hospital stay (14 vs. 18 days, *p* < 0.001). No statistically significant difference was found between the Thymosin-α1 group and control group in duration of symptoms (median, 4 vs. 3 days, *p* = 0.843) and antibiotic utilization rate (14.1% vs. 15.2%, *p* = 0.768).

**Conclusion:** For non-severe patients with COVID-19, Thymosin-α1 can shorten viral RNA shedding duration and hospital stay but did not prevent COVID-19 progression and reduce COVID-19-related mortality rate.

## Introduction

Since 2019, the global pandemic of coronavirus disease 2019 (COVID-19), caused by severe acute respiratory syndrome coronavirus 2 (SARS-CoV-2), has influenced almost all countries worldwide. Although considerable efforts have been made to reduce COVID-19 transmission, the overall upward trend of COVID-19 is continuing around the world. As of 31 January 2021, the outbreak of COVID-19 brings the cumulative numbers to over 102 million reported cases and over 2.2 million deaths globally (1). The disease spectrum of COVID-19 ranges from mild self-limiting disease to severe life-threatening disease, which might progress to acute respiratory distress syndrome, multiple-organ dysfunction syndrome, and death (2, 3).

Immune function dysregulations, including lymphopenia and cytokine storm, were associated with COVID-19 progression (4). Thymosin alpha 1 (Thymosin-α1) is an immune function modifier, which plays an important role in activating and regulating immune cells. Therefore, Thymosin-α1 has been used in diseases with impaired immune function, particularly infections including viral infections (5). In 2003, Thymosin-α1 had been used as an immune enhancer in SARS patients, demonstrating efficacy in controlling the progression of SARS (6). Therefore, Thymosin-α1 has potential as a drug for the treatment of COVID-19 patients.

A recent study showed that Thymosin-α1 reversed T-cell exhaustion and recovered immune reconstitution through promoting thymus output, and then significantly reduced mortality in severe COVID-19 patients (7). Another study also showed that Thymosin-α1 therapy significantly reduced 28-day mortality (HR, 0.11, 95% CI 0.02–0.63, *p* = 0.013) in severe patients with COVID-19 (8). However, the two studies only evaluated the efficiency of Thymosin-α1 on severe patients with COVID-19. To date, there is no available data regarding the efficiency of Thymosin-α1 in non-severe patients with COVID-19. In this study, we aimed to compare clinical outcomes of patients treated with or without Thymosin-α1 therapy in non-severe patients with COVID-19.

## Methods

### Participants

A total of 1,511 consecutive confirmed patients with COVID-19 admitted to the Shanghai Public Health Clinical Center from January 20th 2020 to January 31st 2021 were retrospectively analyzed. The Shanghai Public Health Clinical Center is a tertiary teaching hospital, and the only designated hospital for the treatment of adult patients with COVID-19 in Shanghai, China. Exclusion criteria are as follows: (1) Severe cases requiring immediate intensive care unit (ICU) admission at hospital admission (*n* = 15); (2) using corticosteroid therapy before progression to severe cases (*n* = 63); (3) using intravenous immunoglobulin therapy before progression to severe cases (*n* = 41); and (4) using Thymosin-α1 therapy after progression to severe cases (*n* = 4). Finally, 1,388 non-severe patients with COVID-19 at hospital admission were enrolled.

### Diagnostic Criteria

The following are the diagnostic criteria: collected nasopharyngeal or throat swab specimens of suspected patients with COVID-19, extracted viral nucleotides in specimens, and detected SARS-CoV-2 RNA by reverse transcription polymerase chain reaction (RT-PCR) assay. Patients with COVID-19 were confirmed according to the positive results of SARS-CoV-2 RNA tests. Severe patients with COVID-19 were diagnosed according to at least one of the following standards (9): (1) respiratory frequency ≥ 30 breath/min; (2) resting oxygen saturation ≤ 93%; (3) oxygenation index ≤ 300 mmHg; (4) mechanical ventilation; and (5) shock or other organ failures.

### Details for Standard Therapy

In this study, patients in the Thymosin-α1 group received both Thymosin-α1 therapy and standard therapy, and patients in the control group only received standard therapy. At hospital admission, patients received standard therapy, including oxygen therapy (nasal catheter oxygen inhalation, 3 L/min), antiviral therapy (Traditional Chinese Medicine Decoction, one dose of quaque die; hydroxychloroquine 400 mg quaque die; lopinavir 200 mg/ritonavir 50 mg twice a day; or Arbidol 200 mg three times a day), and allowance of nutrients (three eggs daily, human albumin 10 g quaque die if necessary). During the hospitalization, the oxygen flow rate and drug dosage could be modulated by a joint discussion of at least five experts from the Shanghai Medical Expert Group for the Treatment of COVID-19, based on the change in patients' general conditions, laboratory parameters, and chest CT scans results, and referring to the latest therapy advances in COVID-19.

### Details for Administration of Thymosin-α1

The uses of Thymosin-α1 were decided by a joint discussion of at least five experts from the Shanghai Medical Expert Group for the Treatment of COVID-19, based on patients' age, comorbidity, and laboratorial parameters including lymphocyte count, CD8+ T cell count, and CD4+ T cell count. The dose of Thymosin-α1 and date of administration are shown as follows: (1) 1.6 mg, three times a week, for at least 1 week, 82 patients; (2) 1.6 mg, once every 2 days, for at least 6 days, 94 patients; and (3) 1.6 mg, quaque die, for at least 3 days, 56 patients. Thymosin-α1 therapy was initiated within a median of 2 days (IQR, 1–3) of hospital admission.

### SARS-CoV-2 RNA Extraction Method and PCR Protocol

SARS-CoV-2 nucleic acids were detected using the automatic magnetic extraction device and accompanying kit (Bio-Germ Medical Technology Co., Ltd, Shanghai, China) and screened with a semi-quantitative RT-PCR kits (Bio-Germ Medical Technology Co., Ltd, Shanghai, China) with amplification targeting the ORF1a/b and N gene. The RT-PCR with 5 μL RNA was used to target the nucleocapsid gene and open reading frame lab gene using a SARS-CoV-2 nucleic acid detection reagent (Bio-Germ Medical Technology Co., Ltd, Shanghai, China). The final reaction mixture concentration was 500 nm for primer and 200 nm for probe, respectively. Conditions for the amplifications were 50°C for 15 min, 95°C for 3 min, followed by 45 cycles of 95°C for 15 s and 60°C for 30 s. The lowest detection concentration is 1 × 10^3^ copies/ml.

### Clinical Outcomes and Definitions

In this study, primary clinical outcomes included the rate of patients progressed to severe cases and the COVID-19-related mortality rate. Secondary clinical outcomes included duration of symptoms, SARS-CoV-2 RNA shedding duration, length of hospital stay, and antibiotic utilization rate. In this study, the quantification of the SARS-CoV-2 viral load is not available. Instead, the twice consecutive SARS-CoV-2 RNA negative results with at least 24 h intervals were considered as viral RNA shedding. The SARS-CoV-2 RNA shedding duration was defined as the time from illness onset (symptom onset for symptomatic patients, and first positive SARS-CoV-2 RNA tests for asymptomatic patients) to the occurrence of twice consecutive SARS-CoV-2 RNA negative results with at least 24 h intervals.

### Data Collection

Demographic data including age, sex, body mass index, and comorbidity was obtained. Clinical data including epidemiological histories, clinical manifestations, vital signs, laboratory parameters, chest CT scans results, treatments, hospital stays, and primary and secondary clinical outcomes were collected from electronic medical records.

### Statistical Analysis

Normally distributed data, non-normal distribution continuous data, and categorical data were presented as mean ± standard deviation, median (interquartile range, IQR), and number (frequency), respectively. The statistical difference was compared using Student's *t*-test for normally distributed data, non-parametric Mann–Whitney test for non-normal distribution continuous data, and Chi-square test for categorical data. Propensity score matching (PSM) is a powerful tool for comparing groups with similar observed characteristics without specifying the relationship between confounders and clinical outcomes (10). The PSM method was used to adjust for differences in the baseline data of patients between the Thymosin-α1 group and control group. Propensity scores were estimated according to the essential covariates that might have affected patient assignment to the Thymosin-α1 group or control group, as well as the clinical outcomes of patients with COVID-19. Univariate and multivariable logistic regression analyses were used to identify the covariates that independently associated with primary clinical outcomes. A 1:1 ratio exposed (Thymosin-α1 group) and unexposed (control group) matched analysis was performed; the caliper was set as 0.25 (11). The statistical analyses were performed using the SPSS software, version 15.0 (SPSS Inc. Chicago, Illinois, USA), the MedCalc software, version 16.1 (MedCalc Software bvba, Ostend, Belgium), and the R software, version 3.6.1 (R Foundation for Statistical Computing, Vienna, Austria). All significance tests were two-tailed, and *p* < 0.05 was considered statistically significant.

## Results

### Baseline Characteristics of Patients

Baseline characteristics of patients are shown in Table 1. The median age was 35 years (IQR, 26–47 years); 857 patients (61.7%) were male, and 203 patients (14.6%) had comorbidity. The median white blood cell (WBC), lymphocyte, CD4+ T cell, CD8+ T cell, C-reactive protein (CRP), lactate dehydrogenase (LDH), and D-dimer were 6.0 × 10^9^/L (IQR, 4.8–7.4), 1.6 × 10^9^/L (IQR, 1.2–2.0), 631 cells/μl (IQR, 469–837), 394 cells/μl (IQR, 273–550), 0.5 mg/L (IQR, 0.5–1.5), 189 U/L (IQR, 167–218), and 0.25 ng/mL (IQR, 0.18–0.38), respectively.

**Table 1:** Baseline characteristics of patients.

	**All patients**	**Thymosin-α1 group**	**Control group**	***p*-values**
Number of patients	1,388	232	1,156	–
Age (years)	35 (26–47)	38 (28–53)	34 (26–47)	<0.001
Male, *n* (%)	857 (61.7%)	137 (59.1%)	720 (62.3%)	0.355
Comorbidity, *n* (%)	203 (14.6%)	50 (21.6%)	153 (13.2%)	0.001
**Vital signs**				
Temperature (°C)	37.3 (36.9–37.6)	37.4 (36.8–38.0)	37.2 (37.0–37.6)	0.689
Respiratory rates (/min)	22 (18–24)	22 (18–26)	21 (19–23)	0.571
Heart rates (/min)	75 (68–86)	78 (66–88)	75 (69–85)	0.285
Oxygen saturation (%)	96 (96–99)	96 (95–99)	97 (96–98)	0.369
**Laboratory parameters at admission**				
WBC count (10^9^/L)	6.0 (4.8–7.4)	4.9 (4.0–6.1)	6.2 (5.0–7.5)	<0.001
Lymphocyte (10^9^/L)	1.6 (1.2–2.0)	1.1 (0.8–1.4)	1.7 (1.3–2.1)	<0.001
CD4+ T cell (cells/μl)	631 (469–837)	392 (307–551)	671 (523–875)	<0.001
CD8+ T cell (cells/μl)	394 (273–550)	253 (172–388)	417 (299–583)	<0.001
CRP (mg/L)	0.5 (0.5–1.5)	1.5 (0.5–6.5)	0.5 (0.5–0.8)	<0.001
LDH (U/L)	189 (167–218)	229 (205–258)	175 (160–210)	<0.001
D-Dimer (ng/mL)	0.25 (0.18–0.38)	0.32 (0.23–0.50)	0.24 (0.18–0.36)	<0.001
**Antiviral therapy**				
Chinese medicine	808 (58.2%)	113 (48.7%)	695 (60.1%)	0.001
Hydroxychloroquine	275 (19.8%)	78 (33.6%)	197 (17.0%)	<0.001
Lopinavir/ritonavir	78 (5.6%)	10 (4.3%)	68 (5.9%)	0.343
Arbidol	107 (7.7%)	15 (6.5%)	92 (8.0%)	0.437
Progression to severe cases	12 (0.86%)	4 (1.72%)	8 (0.69%)	0.121

*WBC, white blood cell; CRP, C-reactive protein; LDH, lactate dehydrogenase; p-values indicate differences between the Thymosin-α1 group and the control group*.

Among 1,388 enrolled patients, 232 patients (16.7%) received both Thymosin-α1 therapy and standard therapy (Thymosin-α1 group), and 1,156 patients (83.3%) only received standard therapy (control group). Compared with patients in the control group, those with higher age (38 vs. 34 years, *p* < 0.001), more common comorbidity (21.6% vs. 13.2%, *p* = 0.001), lower WBC (4.9 vs. 6.2 × 10^9^/L, *p* < 0.001), lymphocyte (1.1 vs. 1.7 × 10^9^/L, *p* < 0.001), CD4+ T cell (392 vs. 671 cells/μl, *p* < 0.001), and CD8+ T cell (253 vs. 417 cells/μl, *p* < 0.001) were more likely to be treated with Thymosin-α1 (Table 1).

### Variables Associated With Primary Clinical Outcomes

Variables associated with primary clinical outcomes are shown in Table 2. Univariate analysis showed that age, comorbidity, lymphocyte, CD4+ T cell, CRP, LDH, and D-dimer were associated with primary clinical outcomes (*p* < 0.05). Multivariable analysis identified age (OR = 1.122, 95% CI, 1.033–1.218, *p* = 0.009), comorbidity (OR = 3.117, 95% CI, 1.415–23.425, *p* < 0.001), CD4+ T cell (OR = 0.882, 95% CI, 0.776–0.997, *p* = 0.026), CRP (OR = 1.016, 95% CI, 1.008–1.048, *p* = 0.018), LDH (OR = 1.056, 95% CI, 1.010–1.125, *p* = 0.012), and D-dimer (OR = 1.124, 95% CI, 1.016–1.192, *p* = 0.004) as the variables independently associated with primary clinical outcomes.

**Table 2:** Variables associated with primary clinical outcomes.

	**Univariate analysis**		**Multivariate analysis**	
	**OR (95% CI)**	***p*-values**	**OR (95% CI)**	***p*-values**
Age (years)	1.138 (1.074–1.206)	<0.001	1.122 (1.033–1.218)	0.009
Male	1.033 (0.246–4.340)	0.965		
Comorbidity	18.015 (3.610–89.892)	<0.001	3.117 (1.415–23.425)	<0.001
Fever (T > 37.3°C)	1.193 (0.266–5.350)	0.817		
Respiratory rates (/min)	1.073 (0.747–1.540)	0.704		
Heart rates (/min)	1.013 (0.978–1.048)	0.475		
Oxygen saturation (%)	0.728 (0.170–3.115)	0.669		
WBC count (10^9^/L)	0.930 (0.651–1.328)	0.689		
Lymphocyte (10^9^/L)	0.996 (0.992–0.999)	0.023	0.847 (0.811–1.387)	0.194
CD4+ T cell (cells/μl)	0.775 (0.514–0.904)	0.003	0.882 (0.776–0.997)	0.026
CD8+ T cell (cells/μl)	0.996 (0.992–1.001)	0.098		
CRP (mg/L)	1.041 (1.011–1.072)	0.007	1.016 (1.008–1.048)	0.018
LDH (U/L)	1.100 (1.014–1.204)	0.004	1.056 (1.010–1.125)	0.012
D-Dimer (ng/mL)	1.307 (1.055–1.619)	<0.001	1.124 (1.016–1.192)	0.004
Chinese medicine	0.716 (0.178–2.876)	0.638		
Hydroxychloroquine	1.719 (0.409–7.231)	0.460		
Lopinavir/ritonavir	2.417 (0.294–19.897)	0.412		
Arbidol	2.595 (0.315–21.378)	0.375		

*WBC, white blood cell; CRP, C-reactive protein; LDH, lactate dehydrogenase. Multivariate analysis was fitted by including factors associated with primary outcomes in univariable analyses (p < 0.05)*.

### Characteristics of Patients After PSM

As statistically significant differences existed in the baseline characteristics between the Thymosin-α1 group and control group, we selected patients by the PSM method according to the 1:1 ratio. The factors that independently associated with primary clinical outcomes (age, comorbidity, CD4+ T cell, CRP, LDH, and D-dimer) were matched between the Thymosin-α1 group and control group. After PSM, the baseline characteristics of patients were well-balanced between the Thymosin-α1 group and control group (*p* > 0.05) (Table 3).

**Table 3:** Baseline characteristics of patients after propensity score matching.

	**Thymosin-α1 group**	**Control group**	***p*-values**
Number of patients	184	184	—
Age (years)	37 (28–52)	37 (29–44)	0.193
Male, *n* (%)	105 (57.1%)	109 (59.2%)	0.673
Comorbidity, *n* (%)	37 (20.1%)	35 (19.0%)	0.793
**Vital signs**			
Temperature (°C)	37.3 (36.5–37.8)	37.3 (36.7–37.6)	0.655
Respiratory rates (/min)	21 (18–25)	22 (19–24)	0.469
Heart rates (/min)	76 (65–86)	74 (68–84)	0.841
Oxygen saturation (%)	96 (95–99)	96 (95–99)	0.696
**Laboratory parameters at admission**			
WBC (10^9^/L)	5.1 (4.0–6.1)	5.3 (4.3–6.1)	0.566
Lymphocyte (10^9^/L)	1.1 (0.8–1.5)	1.2 (0.8–1.5)	0.378
CD4+ T cell (cells/μl)	383 (312–568)	378 (308–559)	0.459
CD8+ T cell (cells/μl)	256 (175–390)	254 (180–386)	0.707
CRP (mg/L)	1.5 (0.5–5.7)	1.5 (0.5–5.1)	0.364
LDH (U/L)	218 (198–242)	214 (186–238)	0.620
D-Dimer (ng/mL)	0.31 (0.23–0.48)	0.30 (0.21–0.42)	0.134
**Antiviral therapy**			
Chinese medicine	101 (54.9%)	112 (60.9%)	0.246
Hydroxychloroquine	65 (35.3%)	58 (31.5%)	0.439
Lopinavir/ritonavir	8 (4.3%)	10 (5.4%)	0.629
Arbidol	12 (6.5%)	14 (7.6%)	0.684

*WBC, white blood cell count; CRP, C-reactive protein; LDH, lactate dehydrogenase; p-values indicate differences between the Thymosin-α1 group and the control group*.

### Evaluation of Efficacy for Thymosin-α1

The evaluation of efficacy for Thymosin-α1 in propensity-matched groups is shown in Table 4. The proportion of patients progressed to severe COVID-19 was 2.17% for the Thymosin-α1 group, and 2.71% for the control group (*p* = 0.736). The COVID-19-related mortality was 0.54% for the Thymosin-α1 group and 0 for the control group (*p* = 0.317). Compared with the control group, the Thymosin-α1 group had significantly shorter SARS-CoV-2 RNA shedding duration (13 vs. 16 days, *p* = 0.025) and hospital stay (14 vs. 18 days, *p* < 0.001). No statistically significant difference was found between the Thymosin-α1 group and control group in duration of symptoms (median, 4 vs. 3 days, *p* = 0.843) and antibiotic utilization rate (14.1% vs. 15.2%, *p* = 0.768). In this study, there were no allergic reaction and drug eruption in both Thymosin-α1 group and control group. No significant difference was found between Thymosin-α1 group and control group in liver injury (21.7% vs. 19.7%, *p* = 0.607).

**Table 4:** Evaluation of efficacy for Thymosin-α1 in propensity-matched groups.

	**Thymosin-α1 group**	**Control group**	***p*-values**
Number of patients	184	184	—
**Primary outcomes**			
Developed to severe cases	4 (2.17%)	5 (2.71%)	0.736
Died	1 (0.54%)	0	0.317
**Secondary outcomes**			
Duration of symptom (days)	4 (2–6)	3 (2–5)	0.843
Viral RNA shedding duration (days)	13 (10–19)	16 (11–20)	0.025
Hospital stays (days)	14 (11–21)	18 (13–23)	<0.001
Antibiotics therapy, *n* (%)	26 (14.1%)	28 (15.2%)	0.768
**Probable adverse effects**			
Allergic reaction	0	0	–
Drug eruption	0	0	–
Liver injury	40 (21.7%)	36 (19.7%)	0.607

*Liver injury is defined as ALT > 40 IU/L during the hospitalization*.

### Cox Analysis for Comparison of Time Variables Between Groups

**Figure 1:**
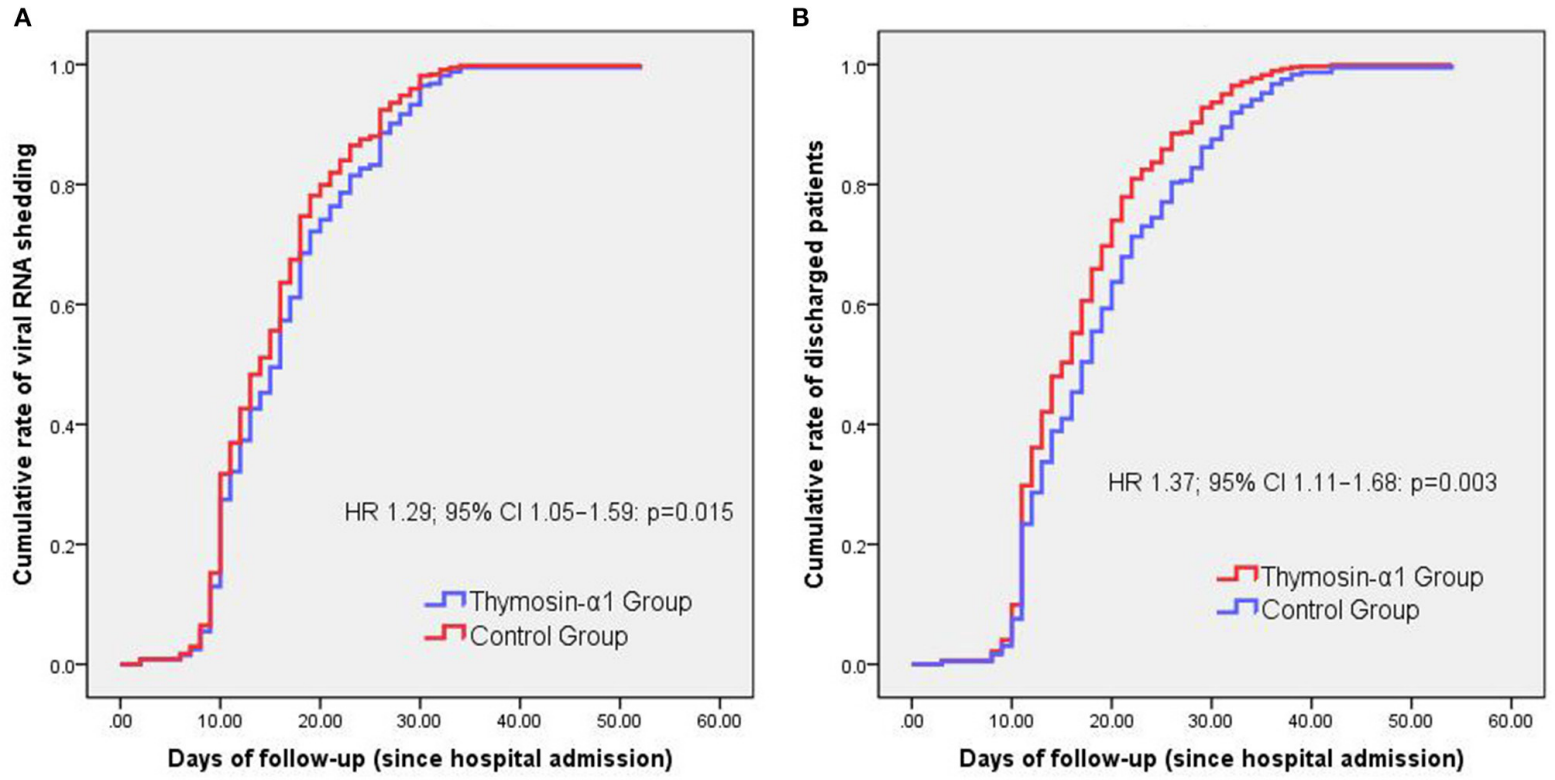
Cox analysis for comparison of time variables between groups. The only one patient who died was excluded when we compared the cumulative rates of discharged patients over days of follow-up between groups. Thymosin-α1 therapy is associated with a shorter SARS-CoV-2 RNA shedding duration (HR 1.29; 95% CI 1.05–1.59: *p* = 0.015) **(A)** and hospital stay (HR 1.37; 95% CI 1.11–1.68: *p* = 0.003) **(B)**, compared with the control group.

Cox regression analysis showed that Thymosin-α1 therapy is associated with a shorter SARS-CoV-2 RNA shedding duration (HR 1.29; 95% CI 1.05–1.59: *p* = 0.015) (Figure 1A) and hospital stay (HR 1.37; 95% CI 1.11–1.68: *p* = 0.003) (Figure 1B), compared with the control group.

## Discussion

Although it is important to explore the potential benefits that Thymosin-α1 can bring in patients with COVID-19, so far, clinical studies on the efficiency of Thymosin-α1 are still limited. In this study that evaluated the efficacy of Thymosin-α1 in non-severe patients with COVID-19, we found that Thymosin-α1 treatment did not alter disease progression and mortality rate, but it significantly reduced SARS-CoV-2 RNA shedding duration and hospital stay. In this study, we compared non-severe patients with Thymosin-α1 therapy to those with standard therapy, rather than a specific drug, since there is as yet no effective drug for non-severe COVID-19 patients.

The duration of SARS-CoV-2 RNA shedding is often considered in determining an appropriate period of isolation as it is often used as a marker of infectivity. Therefore, the importance of shortened duration of SARS-CoV-2 shedding is the public health implications for reducing COVID-19 transmission. It can hardly be denied that the medical resources, especially the number of hospital beds, are insufficient after the outbreak of the COVID-19 epidemic in many countries and areas. Shortening hospital stay is helpful for relieving the pressure on medical resource including the number of hospital beds. Therefore, based on the results that Thymosin-α1 significantly reduced hospital stay and duration of SARS-CoV-2 RNA shedding, we suggested that Thymosin-α1 could be used as a drug for the treatment of non-severe COVID-19 patients.

Thymosin-α1 can boost immune response via activation of T cell proliferation, differentiation, and maturation that is beneficial for virus clearance (12). Therefore, Thymosin-α1 treatment can support patients with low T cell count since it can help boost immunity. The study by Liu et al. recommended COVID-19 patients whose CD8+ T cell count or CD4+ T cell count lower than 400 or 650/μL, respectively, applies Thymosin-α1 injection to improve their immune function (7). Based on our experience and the results of previous studies, we suggested Thymosin-α1 therapy to patients with old age, comorbidity, and reduced lymphocyte, CD8+ T cell, and CD4+ T cell. In this study, patients in the Thymosin-α1 group had higher age, more common comorbidity, lower lymphocyte, CD4+ T cell, and CD8+ T cell count than patients in the control group.

A study by Dominari et al. showed that Thymosin-α1 significantly promoted the proliferation of activated T cells, and this led to a critical prevention of lymphopenia in elderly COVID-19 patients with comorbidity (13). Yu et al. enrolled 25 severely and critically ill patients with COVID-19 and found that patients in the Thymosin-α1 treatment group had a higher number of lymphocytes than patients without Thymosin-α1 treatment (14). Previous studies on severe cases also suggested that treatment with Thymosin-α1 can markedly decrease 28-day mortality and attenuate acute lung injury in critical type COVID-19 patients (7, 8). As a complement to previous studies, we assessed the effect of using Thymosin-α1 as a supportive treatment for non-severe COVID-19 patients. The findings in this study showed that among non-severe COVID-19 patients, Thymosin-α1 therapy significantly reduced hospital stay and the duration of SARS-CoV-2 RNA shedding. In addition, the safety profile of Thymosin-α1 is good and it is virtually devoid of toxicity. Therefore, we suggested that, besides the fact that it should be used on severe cases, Thymosin-α1 could also be used on non-severe COVID-19 patients.

So far, only the nucleotide analog prodrug remdesivir is approved by the US FDA for the treatment of seriously ill patients with COVID-19 (15), although the WHO recommends corticosteroids for the treatment of patients with severe or critical COVID-19 (16). In addition, convalescent plasma is available for use in patients with severe or life-threatening COVID-19 through Emergency Use Authorization (17). However, to date, no effective drugs have been identified to treat non-severe patients with COVID-19. A recent study reported among non-severe patients with COVID-19, treatment with bamlanivimab and etesevimab was associated with a statistically significant reduction in SARS-CoV-2 viral load, compared with placebo (18). In this study, we found that Thymosin-α1 therapy significantly reduced SARS-CoV-2 RNA shedding duration and hospital stay. Compared with bamlanivimab and etesevimab, Thymosin-α1 is more clinically accessible, more inexpensive, and much safer.

In this study, exclusion criteria did not include patients receiving antiviral and antibacterial drugs. We did not exclude patients receiving antibacterial drugs, because secondary clinical outcomes included antibacterial drug utilization rate. We did not exclude patients receiving antiviral drugs, because most of the patients (1,263 patients, 91.0%) in this study received antiviral drugs, including Traditional Chinese Medicine (808 patients, 58.2%), hydroxychloroquine (275 patients, 19.8%), lopinavir/ritonavir (78 patients, 5.6%), and Arbidol (107, 7.7%). In order to eliminate the effects of antiviral drugs on the clinical outcomes, the PSM method was used to adjust for differences in the use of antiviral drugs. After PSM, the use of antiviral drugs including Chinese Medicine (54.9% vs. 60.9%, *p* = 0.246), hydroxychloroquine (35.3% vs. 31.5%, *p* = 0.439), lopinavir/ritonavir (4.3% vs. 5.4%, *p* = 0.629), and Arbidol (6.5% vs. 7.6%, *p* = 0.684) is well-balanced between the Thymosin-α1 group and control group (*p* > 0.05) (Table 3). In this study, multivariable analysis identified underlying disease as one of the variables independently associated with primary clinical outcomes. Although we did not classify underlying disease as an exclusion criterion, the PSM method was used to adjust for differences in the underlying disease.

This study had several limitations. First, although this study showed that Thymosin-α1 has some benefits to non-severe COVID-19 patients, it should be interpreted with caution because of the inherent nature of the retrospective study. More clinical trials are needed to determine the effect of Thymosin-α1 on non-severe patients with COVID-19. Second, in this study, the patient population who progressed to severe COVID-19 or death was small, which made detecting statistically significant differences between groups more difficult for the primary clinical outcomes. Third, in this retrospective study, the SARS-CoV-2 viral load is not available, so we did not know whether Thymosin-α1 treatment can reduce virus titers. Fourth, genetic factors and the presence of some significant SNP in the host are notable factors in the course of COVID-19. Further studies will be needed to confirm the relationship between host genetics and the effect of Thymosin-α1. Fifth, although it is an interesting research point, the difference in viral detection among nasopharyngeal vs. throat swabs in terms of positivity rates and Ct values is unavailable in this retrospective study. However, nasopharyngeal and throat swab specimens from COVID-19 patients have been compared in previous study (19). Vlek reported that combined throat swabs yield a similar sensitivity to detect SARS-CoV-2 as nasopharyngeal swabs and are a good alternative sampling method, despite a lower Ct value for the nasopharyngeal samples (19).

In conclusion, among non-severe patients with COVID-19, Thymosin-α1 treatment did not alter disease progression and mortality rate, but it significantly reduced SARS-CoV-2 RNA shedding duration and hospital stay. No statistically significant difference in duration of symptoms and antibiotic utilization rate were observed between the Thymosin-α1 group and control group. Prospective randomized controlled clinical trials are needed to further assess the clinical benefit of Thymosin-α1 in non-severe patients with COVID-19.

## Data Availability Statement

The raw data supporting the conclusions of this article will be made available by the authors, without undue reservation.

## Ethics Statement

The studies involving human participants were reviewed and approved by Clinical Research Ethics Committee of the Shanghai Public Health Clinical Center. Written informed consent for participation was not required for this study in accordance with the national legislation and the institutional requirements.

## Author Contributions

QL: study concept and design and drafting of the manuscript. CH, LF, WX, WL, and XX: data collection. CH, LF, WX, and QL: analysis and interpretation of data. LC: critical revision of the manuscript. All authors contributed to the article and approved the submitted version.

## Conflict of Interest

The authors declare that the research was conducted in the absence of any commercial or financial relationships that could be construed as a potential conflict of interest.
